# Clique of Functional Hubs Orchestrates Population Bursts in Developmentally Regulated Neural Networks

**DOI:** 10.1371/journal.pcbi.1003823

**Published:** 2014-09-25

**Authors:** Stefano Luccioli, Eshel Ben-Jacob, Ari Barzilai, Paolo Bonifazi, Alessandro Torcini

**Affiliations:** 1 Consiglio Nazionale delle Ricerche, Istituto dei Sistemi Complessi, Sesto Fiorentino, Italy; 2 Joint Italian-Israeli Laboratory on Integrative Network Neuroscience, Tel Aviv University, Ramat Aviv, Israel; 3 Beverly and Sackler Faculty of Exact Sciences School of Physics and Astronomy, Tel Aviv University, Ramat Aviv, Israel; 4 Department of Neurobiology, George S. Wise Faculty of Life Sciences and Sagol School of Neuroscience, Tel Aviv University, Ramat Aviv, Israel; 5 INFN - Sezione di Firenze and CSDC, Sesto Fiorentino, Italy; Indiana University, United States of America

## Abstract

It has recently been discovered that single neuron stimulation can impact network dynamics in immature and adult neuronal circuits. Here we report a novel mechanism which can explain in neuronal circuits, at an early stage of development, the peculiar role played by a few specific neurons in promoting/arresting the population activity. For this purpose, we consider a standard neuronal network model, with short-term synaptic plasticity, whose population activity is characterized by bursting behavior. The addition of developmentally inspired constraints and correlations in the distribution of the neuronal connectivities and excitabilities leads to the emergence of functional hub neurons, whose stimulation/deletion is critical for the network activity. Functional hubs form a clique, where a precise sequential activation of the neurons is essential to ignite collective events without any need for a specific topological architecture. Unsupervised time-lagged firings of supra-threshold cells, in connection with coordinated entrainments of near-threshold neurons, are the key ingredients to orchestrate population activity.

## Author Summary

To which extent a single neuron can influence brain circuits/networks dynamics? Why only a few neurons display such a strong power? These open questions are inspired by recent experimental observations in developing and adult neuronal circuits, as well as by classical debates within the framework of the single neuron doctrine. In this work we identify and present a mechanism which can explain in neuronal circuits, at some early stage of their development, how and why only a few specific neurons can exhibit such power. For this purpose, we consider a standard neuronal network model whose population activity is characterized by bursting behavior. The introduction of a distribution of correlated neuronal excitabilities and degrees, inspired by the simultaneous presence of younger and older neurons in the network, leads to the emergence of functional hub neurons. These critical cells, whenever perturbed, are capable of suppressing network synchronization. Notably, we show that their strong influence on the population dynamics is not related to their structural properties, but to their operational and structural integration into a clique. These results highlight how network-wide effects can be induced by single neurons without any need for a specific topological architecture.

## Introduction

There is increasing experimental evidence that single neuron firing can impact brain circuits dynamics [1]. It has been shown that a single pyramidal cell can trigger whisker deflection [2], drive sensory perception [3] and modify brain states [4]. Similarly, a single GABAergic hub cell can affect collective activity within the developing hippocampal circuitries [5]. In vivo cortical studies have shown that a single extra action potential (AP) can generate a few dozens extra spikes in its postsynaptic targets [6]. Furthermore, a burst of APs, evoked in a pyramidal cell, can propagate through the network activating locally a high fraction of Somatostatine GABAergic cells (a subset of inhibitory neurons) and a few excitatory cells [7]. The capability of single neurons to evoke sparse [6] and network-wide neuronal events [1], [4], [5] in brain circuits can be interpreted within the framework of the single neuron doctrine, firstly postulated on sensorial perception by Barlow in 1972 [8]. According to this doctrine, the spiking of a single neuron in a network has a high functional relevance being able to code very specifically for high level features of abstraction such as concepts. Face selective cells [9] are a typical example of sparse object representation in the brain and of putative grandmother cells [10]. The sensitivity of neuronal networks to small perturbations, such as those introduced by the firing of a single cell, can also find an explanation within the self-organized criticality (SOC) framework [11]. In the last decade, SOC has widely been proposed as the mechanism underlying power law distributions, with characteristic exponents, featuring the size and duration of population events. These distributions have been measured in-vivo and in-vitro experiments on neuronal networks from invertebrates, rodents, monkeys and humans [12]–[15]. The hypothesis underlying the SOC interpretation is that neuronal networks self-organize into a critical state where responses, over temporal and spatial scales of any size (the so-called “avalanches”), can be triggered by small perturbations. Despite the theoretical frameworks above introduced, one of the main open question is how and why only specific neurons can affect the global network dynamics as observed in [2]–[5]. Two main approaches can be foreseen: a “structural-functional” approach [16]–[20], where the specific topology of the network and the connectivity pattern of the cells are responsible for the relevance of the single neuron or a “dynamical” approach, where the single neuron becomes relevant due to the nonlinear evolution of neuronal excitability and synaptic connectivity in the network [21], [22].

A recent computational study on the synchronization properties of a specific neural circuit [23], has pointed out that the level of burst synchrony is a function of both the network topology and the intrinsic dynamics of peculiar neurons, which have a central location in the network graph. This led the authors to conclude that in realistic neuronal systems the choice of a specific topology is not sufficient to induce an unequivocal dynamical behavior in network activity. To further deepen the comprehension of the interplay among cell excitability and synaptic connectivity in promoting network burst synchrony, in this paper we study the effect of single neurons perturbations on the collective dynamics of a network of leaky-integrate-and-fire neurons with short-term synaptic-plasticity [24]. The relevance of these network models for neuroscience have been demonstrated in many contexts ranging from the modelization of working memory [25] to the possibility to perform computation by ensemble synchronization [26]. Although these models have extensively been studied for their capability to generate spontaneous population bursting, little is known about the influence of single cell perturbations on their global dynamics [24].

In order to analyze the population dynamics in a neural circuit at the initial stage of its development, when both mature and young cells are simultaneously present, we consider a random diluted network presenting developmentally inspired correlations between neuronal excitability and connectivity. The presence of these correlations can render the network sensitive to single neuron perturbation of a few peculiar neurons. The coherent activity of the network can be even arrested by removing or stimulating any of these neurons, which are functional hubs arranged in a *clique* regulating the neuronal bursting. We show that the level of available synaptic resources influences the reciprocal firing times of the synaptically connected neurons of the clique. However, the fundamental mechanism responsible for the burst triggering relies on an unsupervised process leading to a precise firing sequence between the neurons which are not structurally connected. Furthermore, frequency locking of the same neurons led, counter-intuitively, to anti-resonances [27], [28], inducing reduced bursting activity or even complete silence in the circuit. Notably, although obtained in a developmentally regulated framework, these results can also be extended to a more general context where the effective connectivity and excitability of the neurons are dynamically regulated by the different states of brain processing.

## Results

In this paper we intend to mimic an immature neuronal network *frozen* at a certain stage of its initial development, similar to the one examined in the experimental work on developmental hippocampal circuits [5] which inspired this work. At early postnatal stages, the main features characterizing such networks are the excitatory action of GABAergic transmission (which is instead the most common inhibitory source in mature circuits) and the presence of synchronized network events, as largely documented in central and peripheral nervous circuits [29]. According to that, we consider a network model composed of only excitatory neurons and displaying bursting activity.

In particular, we considered a directed random network made of 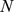 leaky-integrate-and-fire (LIF) neurons [30], [31] interacting via excitatory synapses and regulated by short-term-synaptic-plasticity (see Methods for more details), similarly to the model introduced by Tsodyks-Uziel-Markram (TUM) [24]. As previously shown in [24], [26], [32], these networks exhibit a dynamical behavior characterized by an alternance of short periods of quasi-synchronous firing (*population bursts*, PBs) and long time intervals of asynchronous firing. Notably, the presence of short-term-synaptic-plasticity is the crucial ingredient to observe PBs, even without an inhibitory population [24], [26], [32]. Therefore, the TUM model with excitatory synapses can be considered as a minimal model to mimic the experimentally described stereotypical/characteristic condition of developing neuronal networks [33].

Furthermore, in developing networks, both mature and young neurons are present at the same time, and this feature is reflected in the variability of the structural connectivities and of the intrinsic excitabilities. Experimental observations indicate that younger cells have a more pronounced excitability [34], [35], while mature cells exhibit a higher number of synaptic inputs [5], [36]. Thus suggesting that the number of afferent and efferent synaptic connections [5], [36], [37] as well as their level of hyperpolarization [38] are positively correlated with the maturation stage of the cells. The gradient of excitability - with younger neurons more excitable than older ones - could be explained by a gradient in the excitatory action of GABAergic transmission, i.e. older neurons receive a less depolarizing action by GABAergic input [33].

The presence at the same time of younger and older neurons can be modeled by considering correlations among the in-degree and out-degree of each cell as well as among their intrinsic excitability and connectivity. In particular, in the attempt to find the network organization which is more sensitive to single neuron perturbations, we compare the dynamics of networks where none, one or more of the following correlations have been embedded (for more details see Methods and Supplementary Information):

- setup T1: positive correlation between the in-degree and out-degree of each neuron;
- setup T2: negative correlation between the intrinsic neuronal excitability and the total connectivity (in-degree plus out-degree);
- setup T3: positive correlation between the intrinsic neuronal excitability and the total connectivity (in-degree plus out-degree).

Correlated networks with all possible combinations of the setups T1-T3 have been examined. However, the paper is mainly devoted to the comparison of the properties of the network with correlations of type T1 and T2 (as displayed in Fig. S1) with the completely uncorrelated one, which is a directed Erdös-Rényi graph (see Figs. S3A, S3B). In order to test the possible influence of hub neurons on the network dynamics, also few structural hubs have been added to the network whenever correlations of type T1 were embedded (see Methods and Fig. S1 for more details).

It is important to stress that correlations of type T1 and T2 have a justification on the fact that we consider networks at their developmental stage, as explained above. Furthermore, the correlation of type T2 can also represent a homeostatic regulation of the neuronal firing to cope with different levels of synaptic inputs [39].

For clarity reasons, the paper will mainly deal with a specific realization of a network, made of 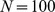 neurons and embedding correlations of type T1 and T2. However, we have verified the validity of our findings in other five realizations of the network with correlations T1 and T2: four for 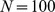 (examined in Text S2) and one corresponding to 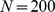 (discussed in details in Text S3).

### Single neuron stimulation/deletion impacts population bursting in developmentally correlated networks

**Figure 1:**
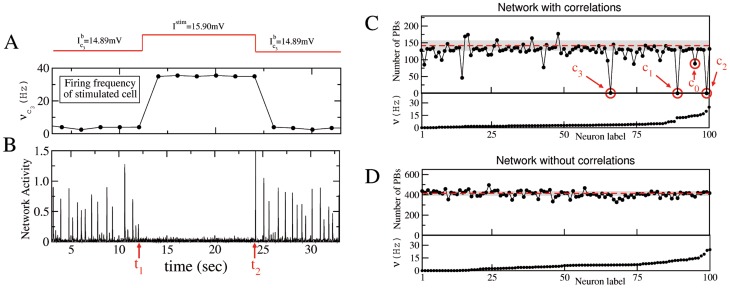
Single neuron stimulation (SNS) can stop population bursting activity in presence of type T1 plus T2 correlations. A sketch of a SNS experiment for a network with type T1 plus T2 correlations is reported in (A) and (B): the neuron 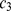 is stimulated with a DC step for a time interval 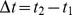 (as shown by the red line on the top panel). Average firing rate of neuron 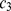 (A) and network activity (B) as measured during the experiment. (C) and (D) refer to correlated and uncorrelated networks, respectively. Upper panels display the number of population bursts, PBs, delivered during SNS experiments versus the stimulated neuron, ordered accordingly to their average firing rates 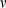 under control conditions (bottom panels). Each neuron 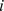 was stimulated with a DC step (switching its excitability from 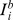 to 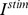) for an interval 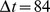 s. The critical neurons are signaled by red circles. The number of PBs, emitted in control conditions within an interval 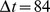 s, are also displayed: red dashed lines indicate their averages, while the shaded gray areas correspond to three standard deviations. The data refer to 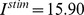 mV and 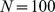 neurons.

In the developing hippocampus it has been shown how the stimulation of specific single neurons can drastically reduce the frequency of the PBs [5], [16]. These neurons have been identified as *hub cells* for their high degree of functional, effective, and structural connectivity [40]. Stimulation consisted of phasic or tonic current injection capable of inducing sustained high firing regime of the stimulated neuron over a period of a few minutes. Based on this experimental observations, we tested the impact of prolonged *single neuron stimulation* (SNS) on the occurrence of PBs on our network model. SNS was obtained by adding abruptly a DC current term to the considered neuron. For illustrative purpose, we report in Fig. 1 A–B the stimulation protocol for a specific neuron capable of suppressing the occurrence of PBs for all the duration of the SNS (in this case limited to 12 s). During the current stimulation the neuron fired with a frequency of 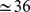 Hz well above the average (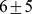 Hz) and the maximal (24 Hz) firing rate of the neurons in the network under control conditions (see the bottom panel in Fig. 1 C).

The stimulation process was completely reversible and after the end of the SNS both the firing rate of the cell and the PBs frequency returned to the pre-stimulation control level. In order to evaluate the impact of single neuron perturbation on the collective dynamics, we considered the variation of PB frequency relative to control conditions (i.e. in absence of any stimulation). In Figs. 1 C,D the impact of a single neuron stimulation on the PBs frequency is reported for a classical Erdös-Rényi network (no correlations) and a network with embedded correlations T1 plus T2. Please notice that the neurons in Figs. 1C–D are ordered according to their average firing rate 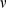 under control conditions, which covered the interval 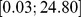 Hz. The comparison of panels C and D in Fig. 1 clearly shows that the correlated network is much more sensitive to single neuron stimulation. In fact, the SNS was able, for three neurons, to suppress the occurrence of PBs during their stimulation, while for approximately another half dozen of neurons the PBs were halved with respect to control conditions. The three most critical neurons 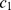, 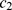 and 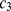 were characterized, before stimulation, by firing frequencies larger than 3.3 Hz, and they lay among the top 33% fastest spiking neurons. On the contrary, in a network where no correlations were present, the SNS had only extremely marginal influence on the population activity (see Fig. 1D), although the firing rate distributions in the correlated and uncorrelated network were extremely similar (under control conditions) as shown in the bottom panels of Fig. 1 C and D.

**Figure 2:**
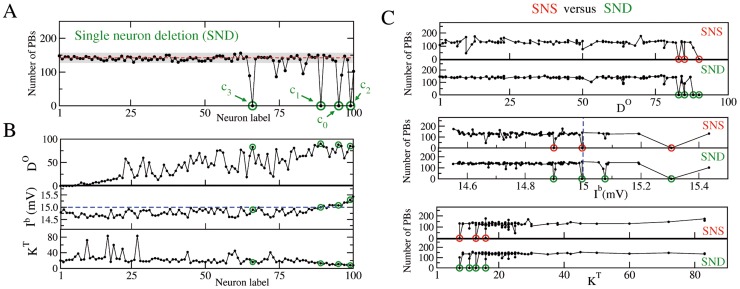
Comparison between single neuron stimulation (SNS) and deletion (SND) in a network with correlations of type 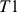 plus 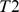. (A) Number of PBs emitted during SND experiments versus the label of the removed neuron. (B) Functional and structural properties of the network, as measured in control conditions, i.e. in absence of any stimulation/manipulation of the neurons. From top to bottom: functional out-degree 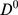, intrinsic excitability 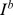, and total structural connectivity 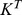. The red dashed line and the gray shaded area in (A) as well as the neuron labels are as in Fig. 1 C, the blue dashed line denotes 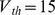 mV. (C) Comparison between SNS and SND: the number of PBs occurring during SNS (resp. SND) is reported as a function of 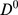, 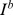 and 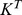. In all panels the green (red) circles mark the critical neurons, which under SND (SNS) can silence the bursting activity of the network. The bursting activity is recorded over an interval 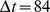 s.

In [24] it was shown that the elimination of a pool of neurons from an uncorrelated network encompassing short-term synaptic plasticity caused a strong reduction of the population bursts. In this work we repeated such numerical experiment with single cell resolution, i.e. we considered the influence of *single neuron deletion*, SND, on the neuronal response of the network (results reported in Fig. 2). For the network with correlations 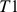 plus 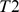, in four cases the SND led to the complete disappearance of PBs within the examined time interval, while for five other neurons their individual removal led to a decrease of the order of 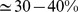 in the frequency of occurrence of the PBs (Fig. 2 A). Three among these four critical neurons (namely, 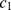, 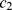 and 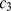) were also responsible for silencing the network during the SNS experiment performed with a stimulation current 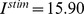 mV, as shown in Fig. 1C and Fig. 2 A. The same SNS experiment on the fourth critical neuron, labeled 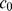, reduced the PB frequency of about 40% with respect to control conditions (Fig. 1C). However, as we will show in the following, a SNS experiment performed on 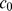 with a different injected current can also lead to a complete silence in the network. Notably, in analogy with the SNS, also this additional critical neuron 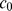 impacting the PB occurrence under SND lays among the top 33% fastest spiking neurons. Differently from SNS, the removal of neurons with lower frequencies had almost no impact on the network dynamics. For uncorrelated networks the effect of SND was almost negligible, inducing a maximal variation in the bursting activity of the order of 10–15% with respect to the activity under control conditions (see Fig. S3C).

#### Other correlation setups

We have also analyzed the response to SNS and SND experiments in networks embedding all the possible combinations of the correlations setups T1-T3. In particular, we considered networks with positive correlation between structural in-degree (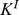) and out-degree (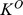) (setup T1 shown in Figs. S4A, S4B), with negative correlation between excitability 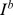 and total connectivity 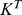 (setup T2 shown in Figs. S5A, S5B), with only positive correlation between 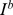 and 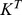 (setup T3 shown in Figs. S6A, S6B) and finally combining positive correlations between 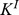 and 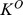 and 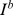 and 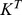 (setups T1 plus T3 shown in Figs. S7A, S7B).

As we are looking for strong impacts on the network dynamics, we identified the network as “sensitive” to SND (or SNS) whenever the PBs frequency was altered more than 90% with respect to the corresponding PB activity in control conditions. Therefore, we considered as significative only the modifications of the activity which were well beyond the statistical fluctuations in the population bursting, shown by the shaded gray area in panels C and D in Figs. S4, S5, S6, and S7.

In all the examined cases, despite the fact that the firing frequencies distributions were quite similar to the ones measured in the correlated network embedding setups T1 and T2, we did not observe significant modifications of the bursting activity by performing SNS and SND experiments on any neuron of the network (see panels C and D in Figs. S4,S5,S6,S7). The situation where SNS and SND had a larger effect on the network activity was for the correlations of type T2. In that specific case we observed that SND on 2 neurons (lying among the top 33% fastest spiking neurons, namely 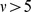 Hz) halved the bursting frequency of the network, and SNS on one of these 2 neurons had a similar effect. In all the other cases the PB activity was never perturbed more than 20–30%. Only the simultaneous presence of type T1 and type T2 correlations noticeably enhanced the sensitivity to SNS and SND, leading to the possibility to silence the network.

### Structural and functional properties of the network

In order to gain some insight into the mechanisms underlying the reported response of the network, with correlations of type 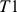 plus 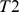, to SNS and SND experiments, we analyzed the structural and functional connectivity of the network in relation to the intrinsic excitability of the neurons. Functional connectivity (FC) analysis [41] was aimed at revealing time-lagged firing correlations between neuronal pairs, similarly to what described in [5] for the developing hippocampus. In particular, for every possible pairs of neurons 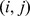 we cross-correlated their spike time series, with the exclusion of the spikes occurring within bursts, for which only the timestamp of the first spike was kept (see Methods). A functional connection directed from 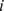 to 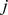 was established whenever the activation of 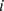 reliably preceded the activation of 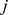 and viceversa (see Methods). For each cell 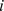, we calculated the functional out-degree (in-degree) 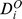 (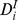), i.e. the number of cells which were reliably activated after (before) its firing.

As shown in the top panel of Fig. 2B, the four critical neurons, 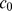
–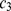, identified during the SNS and SND experiments, have very high functional out-degree, namely 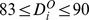. In particular, three of them (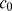, 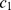 and 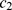) are ranked among the first four neurons with the highest functional out-degree. Therefore the critical neurons are reliably preceding the activation of most of the other neurons in the network. In addition, neurons 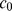 and 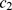 were supra-threshold (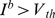, see Methods) and therefore firing tonically even if isolated from the network, while neuron 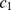 was at threshold and 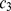 below it (as shown in the central panel of Fig. 2B).

In contrast to their high functional out-degree, critical neurons were characterized by a low structural degree 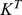 (total number of afferent and efferent connections), namely 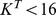 with respect to an average value 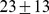, as shown in the bottom panel of Fig. 2B. This result was a direct consequence of the anti-correlation imposed between total degree and excitability and this represented a crucial aspect for the emergence of the critical neurons.

In Fig. 2 C we report the results of SNS (SND) experiments as a function of 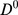, 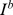 and 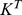 of the stimulated (removed) neurons. The experiments on the neurons with high 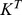 (the structural hubs, shown in Fig. S1 A and S1 B) influenced marginally the network bursting, apart for the single neuron stimulation of the two principal hubs which led to a moderate increase of the activity (see the bottom panels in Fig. 2 C). However SND on the same neurons had no significant effect. On the other hand, neurons with high functional out-degree 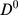 (functional hubs) were quite relevant to sustain the collective dynamics. The removal of neurons with low 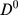 (including the structural hubs) seemed almost not affecting the bursting properties of the network. Altogether, apart the stressed differences, the SNS and SND experiments appeared to give quite similar results.

The generality of these findings have been tested by performing SNS/SND experiments on other five different realizations of the network with embedded correlations of type T1 and T2, in all cases a small subset of neurons resulted to be critical in the sense discussed above (for more details see Text S2 and S3).

### Network response during SNS: Dependence on the injected current

**Figure 3:**
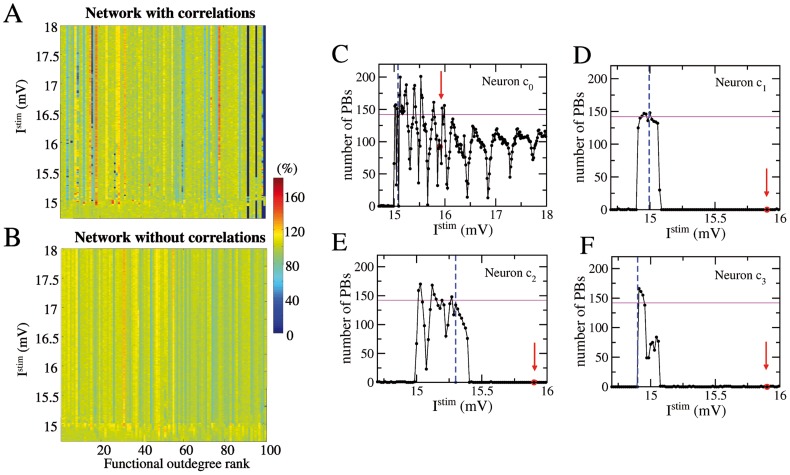
Impact of single neuron stimulation on the population activity: dependence on the injected current. Color coded rates of emission of PBs during SNS experiment performed on each single neuron for a range of injected DC currents 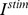 (y-axis) in networks with correlations of type T1 plus T2 (A) and without any correlations (B). The neurons are ordered according to their functional out-degree rank (x-axis) and the PB rates during SNS are normalized to the PB rate in control conditions. (C–F) Number of PBs emitted during SNS of the critical neurons 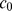,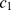,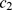, 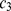 versus the stimulation current 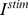. The red arrows indicate 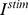 employed for the SNS experiments in Fig. 1 C. The blue vertical dashed lines mark the value of the intrinsic excitability and the horizontal magenta solid line the bursting activity of the network, both measured at rest. The number of PBs are measured over a time interval 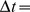 84 s.

In order to further clarify the impact of varying the intrinsic excitability of single neurons on the network bursting activity, we have performed extensive analysis of the network response under SNS experiments for a wide range of stimulation currents, namely 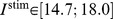 mV. In panels A and B of Fig. 3 it is summarized the impact on the bursting activity of the SNS for networks with type T1 and T2 correlations and without any correlations. SNS had really a minimal effect on the uncorrelated network: in this case the number of emitted PBs varied only up to a 20% with respect to control conditions. On the contrary, for the correlated network, SNS was able to silence the network over a wide range of currents when 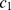, 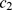 and 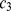 were stimulated. For the other neurons, SNS with high stimulation currents could also have the effect of promoting an increase of PBs up to 130–140% with respect to control conditions. In particular, an increase in the PB activity has been observed consistently for two structural hubs, whenever they are brought above the firing threshold, as shown in Fig. S1 C, and for other two neurons directly connected to these hubs. This behaviour is expected for an excitatory network without correlations, where the neurons with higher out-degree have usually the highest impact on the network [19]. However, the removal of each of these four neurons from the network did not influence the PB activity, furthermore they were passively recruited during bursting events.These results had an explanation in the fact that in control conditions the structural hubs were well below threshold, due to the anti-correlation between total degree and excitability, while by increasing the stimulation on these hubs we violated such constraint.

As shown in panels D, E, F of Fig. 3, for neurons 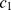, 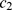, and 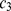 the bursting activity survived only in narrow stimulation windows located around, or just above the firing threshold value. A current variation 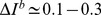 mV was, for these three neurons, sufficient to silence the network. The stimulation of the neuron 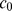, the one critical for SND but not for SNS (when 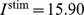 mV), revealed the existence of very narrow *anti-resonance* windows (i.e. minima in the number of emitted PBs), as shown in Fig. 3 C. For very specific intrinsic excitability this neuron could effectively silence the network, but for generic excitation its influence on PBs activity was limited. The anti-resonances occurred (for 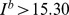 mV) at almost regular intervals: initially of width 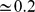 mV and, at larger intrinsic excitability, of width 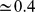 mV. This point will be further discussed and clarified in Sect. *Time Orchestration*.

### The functional clique

**Figure 4:**
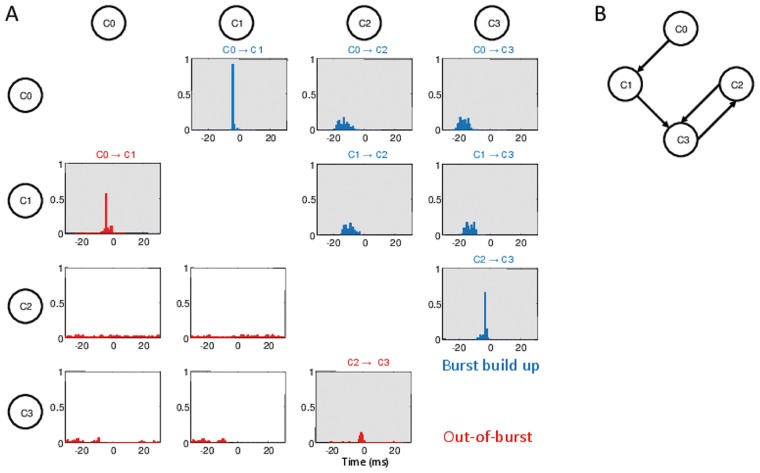
The functional clique. (A) Cross correlation functions C(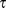) between the spike trains of two critical neurons. 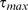 has been measured as the position of the maximum of the cross correlation between the time series of the two considered neurons. The panels refer to all the possible pair combinations of the critical neurons, furthermore blue (red) histograms refer to the analysis performed during the population burst build up (during periods out of the bursting activity). For more details see the subsection Functional Connectivity in Methods. The order of activation of each pair is reported on the top of the corresponding panel, whenever the cross-correlation has a significant maximum at some finite time 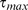. Note that during the PB onset, neurons activate reliably in the following order 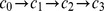. During the out-of-burst activity, clear time-lagged activations are present only among the pairs 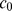-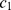 and 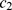-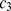. (B) Structural connections among the four critical neurons: the black arrows denote the directed connections. The data here reported, as well in all the following figures, refer to a network with correlations of type T1 plus T2.

**Figure 5:**
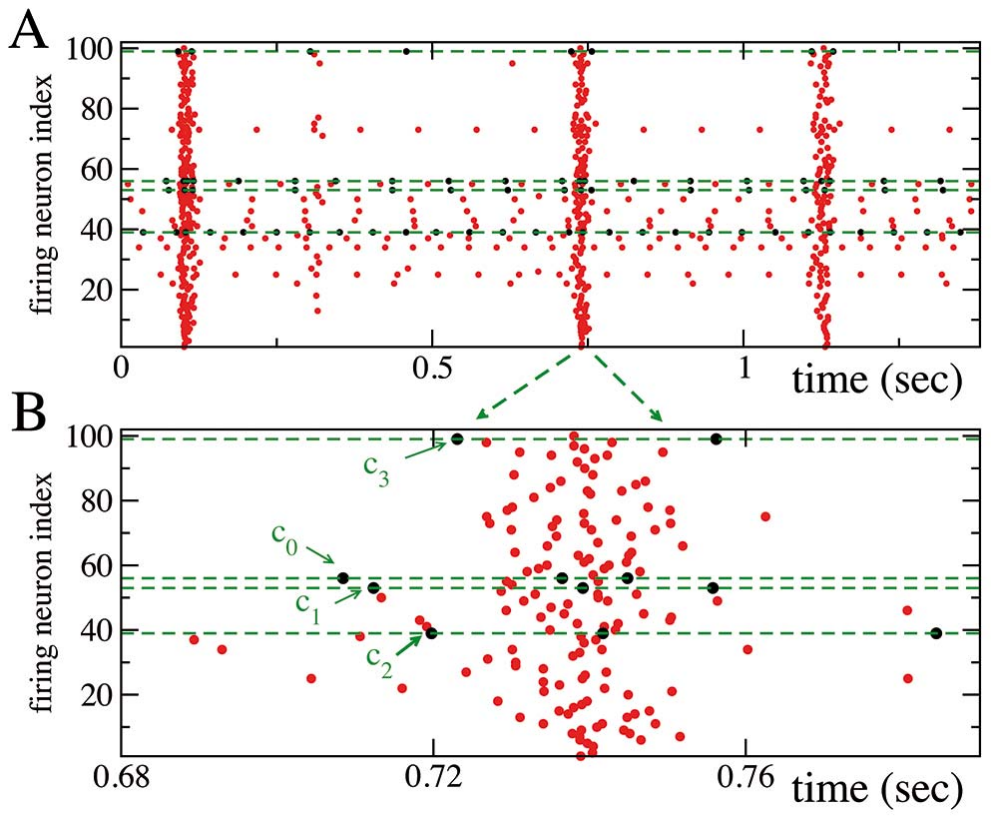
The critical neurons precede the population bursts in a network with correlations of type 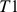 plus 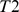. (A) Raster plot of the network activity: every dot denotes a firing event. The dashed green lines and black dots refer to the four critical neurons. (B) Enlargement of a representative population burst: PBs are anticipated by the ordered firing sequence 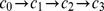. For clarity reasons, in the raster plots, at variance with all the other figures, the neuronal labels are not ordered accordingly to their firing rates.

The results reported above suggest that the four critical neurons 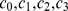, identified in the network with correlations T1 plus T2 should have a key role in the onset of the collective bursting. Therefore, we focused our analysis on the PB build up, i.e. we examined the events occurring in a time window of 25 ms preceding the peak of synchronous activation (for more details see Methods). In particular, we quantified how many times each single neuron participated in the build up of a PB. As we have verified, for the correlated network all the bursts were preceded by the firing of the four critical neurons, while in absence of correlations there was no neuron capable of reliably preceding every burst activation. The cross correlations between the timing of the first spike emitted by each critical neuron during the PB build up (see Methods) are shown in Fig. 4 A (blue histograms). This analysis revealed a precise temporal sequence in the neuronal activation, respectively 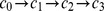, as shown also for a few representative bursts in Fig. 5 A,B (therefore the labeling assigned to these neurons). Interestingly, the same neurons did not show this precise temporal activation out of the PBs, as revealed by the red histograms in Fig. 4 A (see also Methods). Furthermore, the time sequence of the firing events of the critical neurons during the build up of the PB was quite well determined: 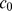 anticipated the firing of 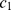 of 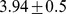 ms, 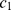 anticipated 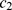 of 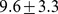 ms and 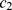 anticipated 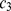 of 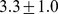 ms. During the inter-burst periods we observed clear time lagged correlations only for the pair 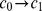, presenting a direct synaptic connection, and in a weaker manner also for the pair 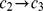. On the basis of the reported data, we can safely affirm that the critical neurons form a *functional clique* responsible for the onset of the PBs.

### The role of plasticity

As clarified in [24], the bursting activity was due to the short-term-synaptic depression. In particular PB emission could be related to the evolution of the fraction of synaptic resources in the recovered state, characterized by the variable 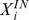 (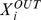), averaged over the afferent (efferent) synapses of each neuron 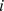 (see Methods). The authors in [24] have shown that the fraction of synaptic resources, averaged over all the excitatory synapses, had a deep minimum in correspondence of the burst event and then slowly recovered its stationary value over a time scale dictated by the average recovery time 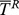. This means that the average effective strength of the excitatory connections (measured by 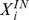 and 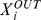) was strongly depressed after a burst, and this inhibited the prosecution of the bursting activity, which could restart only when the strengths of the synapses would return to their stationary values.

**Figure 6:**
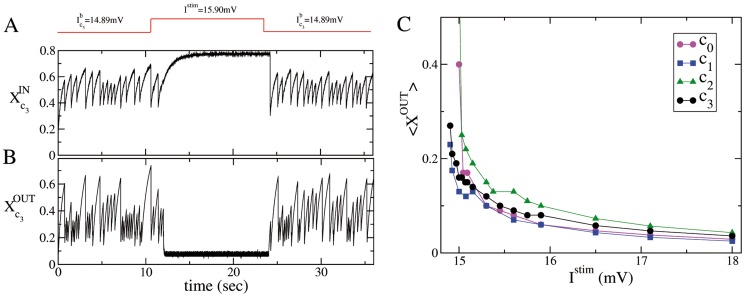
Effective synaptic strength during single neuron current stimulation. Average synaptic strength of the afferent (A), and efferent (B) connections of the critical neuron 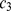 during SNS with 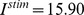 mV (these data corresponds to the experiment reported in Fig. 1). The output (input) effective synaptic strength is measured in terms of the average value of the fraction 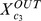 (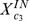) of the synaptic transmitters in the recovered state associated to the efferent (afferent) synapses (see Methods). (C) Time averaged synaptic strengths 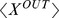 as measured during SNS experiments performed on each of the four critical neurons for various stimulation currents 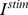. The legend clarifies to which neuron corresponds the average synaptic strengths displayed in the figure, the averages have been performed over 84 s.

In this Section, we want to address the question whether the variation of the effective strength of the synapses could be also responsible for the silencing of the network (with correlations of type T1 plus T2) during SNS experiments. So far we have clarified that the removal of any of the four neurons in the functional clique blocked the bursting activity, however it is not clear why a small stimulation of 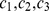 was capable also of blocking the PBs. As reported in Fig. 6 A, B the stimulation of neuron 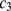 with a large current 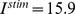 mV (as in the experiment reported in Fig. 1 A,B) reduced noticeably 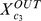, due to the high firing activity of the stimulated neuron. Analogous results have been found for all the other three critical neurons. For neurons 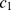, 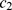 and 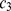 this stimulation blocked the bursting activity of the network, thus inducing an almost complete recovery of the available resources of the afferent synapses, measured by 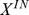 (as shown in Fig. 6 A for 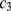). These results could suggest that SNS and SND experiments are indeed equivalent, since if the efferent synapses are extremely depressed, this could correspond somehow to remove the neuron from the network. However, SNS of neuron 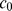 did not lead generically to the suppression of the bursting activity even if its efferent synapses were similarly depressed (as shown in Fig. 3 C). Furthermore, the synaptic depression could not explain the anti-resonances in the bursting activity observed for SNS of 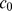 and 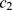 with different 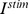. Since the time averaged synaptic strength, 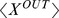, exhibited only a smooth decrease as a function of 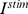 for all the four critical neurons (as well as for any generic neuron in the network), as shown in Fig. 6 C.

### Time orchestration

As already mentioned, the roles of the four neurons in the functional clique of the network with type T1 and T2 correlations were quite well established, and just a precise firing time sequence could induce the population avalanche. To better understand the role of each critical neuron, it is necessary to point out that, under control conditions, the neurons 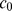 and 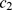 could fire even if isolated (since 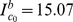 mV and 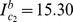 mV were larger than 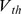), 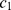 was at threshold (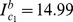 mV) and 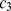 was the only neuron below threshold (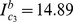 mV). This clearly explains, given the existing synaptic connection from 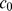 to 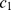 (see Fig. 4 B), the reason why 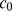 entrained 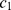, both during the burst build up as well as during the inter-burst periods (see Fig. 4). Furthermore, from the results of the SNS experiments performed on 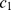 and 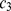 (Panels D and F in Fig. 3) one can observe that the network activity arrested whenever 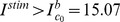 mV for both these neurons (for comparison, note that the range of 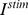 reported in panel C is different from panel D,E and F). Therefore, whenever these two neurons fired faster than the clique leader 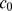, the burst activity, which should be triggered by a well determined sequence of events, would be terminated. Thus we can conclude that 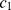 and 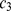 could only be the followers of the dynamics dictated by the two supra-threshold neurons, and in particular by the leader 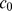.

**Figure 7:**
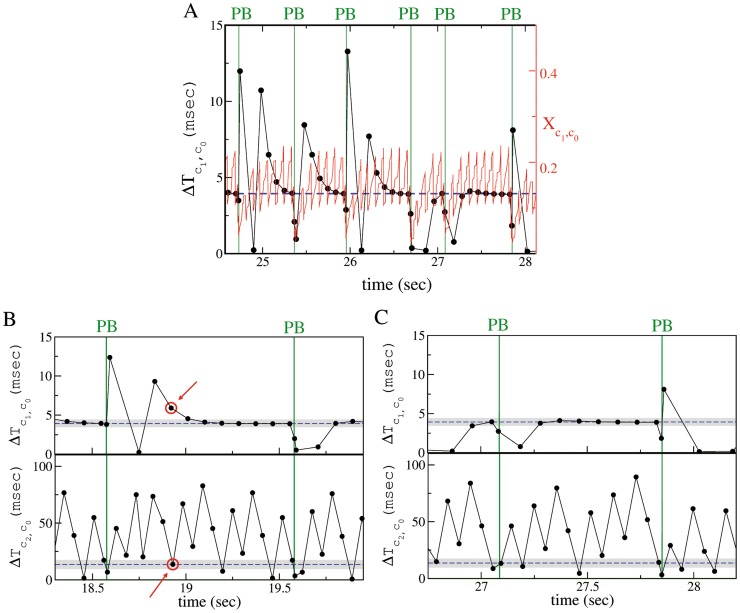
(A) Synapse strength and firing time delay between the neurons 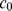 and 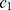. Time evolution of the effective synaptic strength 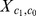 (red solid line and right *y*-axis) and of the firing time delay 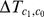 (black line with dots and left *y*-axis). **(B),(C) Failures and successes in population burst ignition.** Spike time delay 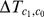 (top panel) and 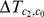 (bottom panel) of neuron 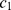 and 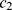, respectively, referred to the last firing time of 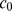. Panels (B) and (C) clearly show that PBs (denoted by green vertical lines) can occur only when the neuron 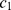 and 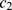 fire within precise time windows after the firing of neuron 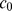. In (B) a clear failure is indicated by red circles, in this case 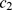 fired at the right time, but 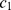 was too slow; in (C) neuron 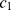 fires at the right moment several times (black dots are within the gray shaded area in the top panel), but the avalanche is not initiated until 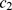 does not emit a spike within a precise time interval after the firing of 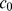. In all the figures, the data refer to control conditions. The blue horizontal dashed lines refer to the average value of 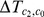 or 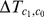 at the PB onset, while the shaded gray areas indicate the corresponding standard deviations.

As clearly shown in Fig. 7 A, exactly before a burst event (i.e. in the PB build up phase) neuron 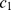 fired with a precise time lag after neuron 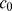 (blue dashed line in the figure). However, the time lag 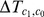 between the firing of 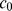 and 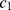 needed some time after each bursting event to adjust to its pre-burst value. This could be interpreted also as an effective refractory period needed to the pair 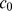-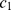 to recover the proper entrainment favorable to the burst discharge. As shown in Fig. 7 A, the time evolution of the variable 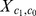, which measured the effective strength of the synapse connecting 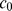 to 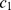, is directly connected to the duration of the time interval 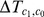 (or analogously to the effective refractory time of the entrainment 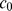-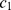). After a burst, 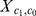 was noticeably depressed (reaching almost zero) and it slowly recovered its asymptotic value over a time scale dictated by 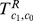. Indeed 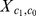 was strongly oscillating due to the firings of 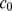, however the recovery of the pre-burst condition can be assessed by considering its extreme values (minima and maxima) both slowly increasing after the burst. The recover of the effective synaptic strength was associated to the adjustment of 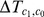 to the value taken during the build up of a PB. From Fig. 7 A, it is also evident that the fulfillment of this condition was not sufficient to induce another PB, since the PB could occur even a long time after the favorable pre-burst value was reached by 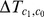.

Similar behaviors had been observed also for the synapse connecting 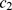 to 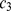, although the firing of neuron 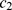 alone was not sufficient to bring 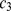 above threshold and therefore to initiate the PB. Indeed, the activation of 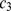, whose firing was fundamental to trigger the avalanche, was more complex. From a structural point of view, the neuron 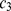 received inputs directly from 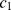 and 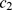, while there were no synaptic connections between 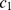 and 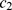 (see Fig. 4 B). The entrained firing of the pair 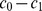 followed by the firing of 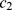, within a precise time window, was required to induce 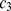 to emit a spike and therefore a PB. This can be clearly appreciated from Fig. 7 B and C. In particular, in Fig. 7 B is reported a situation where 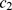 fired at the right time after 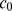, but 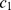 has fired too late to start an avalanche in the network (as previously explained the firing of 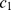 was not yet entrained to that of 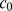). Much more common is the situation reported in Fig. 7 C, where 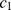 fired essentially always at the same time after 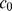, but instead the time delay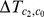 in the firing of 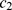 was extremely variable ranging from an almost coincidence with 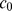 to a delay of 100 ms. The PB could occur only when 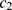 fired in a precise time window following the activation of 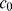. Once noticed that the most part of the PB failures are due to 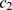 and in a first attempt to understand the emergence of bursts in the network, we can focus only on the firing times of neuron 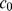 and 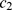.

To get a deeper insight on this issue, let us consider the anti-resonances (corresponding to minima in the PB activity) observed during the SNS experiments performed on 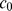 (see Fig. 3 C). To interpret such minima we examined the firing periods 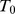 and 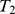 of the neuron 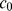 and 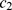 once isolated from the network. For the LIF model [30] these are simply given by 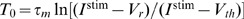 and 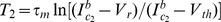, where 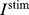 is the stimulation current acting on 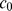 and 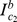 the intrinsic excitability of 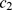. As shown in Table 1 the PB minima were associated to rational ratios of these periods. This amounts to exact frequency locking of the firing of the two neurons [42], whenever this occurs the bursting activity is depressed or even suppressed. This because the build up of a burst relies on a precise temporal mismatch between the firing of neuron 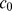 and 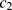, which, in the case of exact locking, can be achieved quite rarely or even never. Therefore, given the absence of any structural connection among these two neurons, the clique functionality relied on unsupervised coordinated firing of 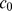 and 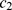.

**Table 1:** Anti-resonances observed during SNS of 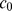.

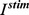 (mV)	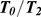	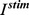 (mV) (Model)
15.04	2	15.04
15.09	8/5	-
15.31	1	15.30
15.48	4/5	15.53
15.66	2/3	15.65
15.88	0.565	-
16.05	1/2	16.03
16.44	2/5	16.43
16.84	1/3	16.83
17.28	0.282	-
17.73	1/4	17.65

**Figure 8:**
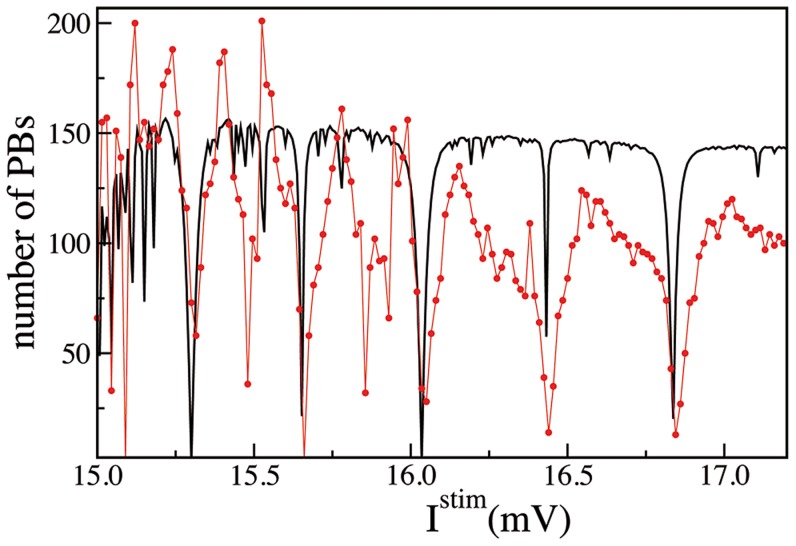
Model based reconstruction of the SNS experiment for the critical neuron 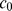. Number of emitted PBs as a function of the stimulation current 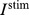 applied to the neuron 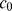. The red line with dots refers to the results of the SNS experiment on 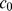 (same curve as in Fig. 3 C) and the black line to the estimations obtained by measuring the PB occurrence with the simple model for SNS, described in the Methods. The measurement were performed in both cases over a time interval 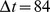 s.

The first column reports the stimulation currents 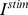 for which pronounced minima (anti-resonances) are observed in the stimulated PB activity during SNS experiment on 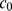 (same data as in Fig. 3 C and red curve in Fig. 8), the second column the corresponding 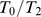 ratios. 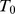 and 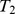 are the firing periods of the LIF neurons 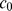 and 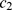 in isolation, namely, 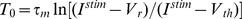 and 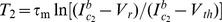. The third column refers to the anti-resonances generated by employing the simple model for SNS of 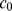 introduced in the Methods (same data as the black curve in Fig. 8). The reported values correspond to the minima in the PB activity for this model, the absence of a value means that the model did not display a corresponding minimum. The data refer to SNS experiments performed over a time interval of duration 84 s.

In order to confirm this hypothesis, we developed a simple model to reproduce the results of the SNS experiment on 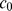. In particular, we assumed that 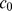 and 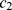 could be considered as two independently spiking neurons with their own firing periods determined by the stimulation current 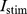 for 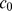 and by the intrinsic excitability for 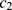. Furthermore, we assumed that a PB is emitted with a certain probability (related to the synaptic depression induced by the stimulation) whenever 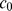 and 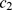 fired in the correct order and with a prescribed time delay (for more details see Methods). The results are reported in Fig. 8 and in the Table 1, the agreement is quite surprising due to the limited ingredients employed in the model. Furthermore, the fact that more than the 60% of the “anti-resonances” as well as the level of the PB activity were reproduced was a clear indication that the simple ingredients at the basis of the model represented the main mechanisms behind the PB build up process in this network. These mechanisms could be summarized as follows: the functional clique can be assumed to be composed of two structurally connected pairs 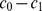 and 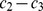, where 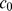 and 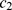 fired tonically and independently one from the other. Any spike emission of 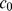 induced a firing of 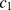, however to recruit 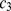 and therefore to initiate the PB, also 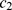 should deliver a spike, with the right time delay after 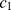. Therefore, if 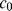 and 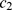 fired with periods which were rational multiples one of the other it was unlikely to build up the PB. Since the synchronism among the two neurons did not allow 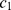 to participate to the build up of the PB. The spike delivered by 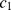 is fundamental to lead 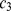 above threshold and to trigger the avalanche, but it should be emitted at the right moment, as clearly shown in Fig. 7 B and C.

## Discussion

The aim of the present work was to identify neuronal network arrangements sensitive to single neuron perturbations, such as those induced by single neuron stimulation or deletion (or forced silencing). We choose as a benchmark model a random network of excitatory LIF neurons, connected via depressive synapses regulated by the TUM mechanism [24]. Such networks displayed spontaneous bursting activity also in absence of inhibition, as extensively described in the literature [24], [26], [32], [43]. The choice of random topology was aimed at revealing the role of developmentally regulated neuronal excitability and connectivity gradients [5], [35]–[38], rather than specific topological configurations, in rendering network organization sensitive to single neuron perturbations.

The introduction of a positive correlation between in- and out-degree (T1) and a negative correlation between intrinsic neuronal excitability and total degree (T2), besides being justified from a developmental point of view, favors also the stabilization of the network activity. This because, as pointed out in [19], in an excitatory network the sensitivity to fluctuations is mainly due to cells with a high out-degree. Therefore, to avoid that their activation during spontaneous activity can cause network destabilization, a possible strategy is to impose an anti-correlation between their level of excitability and their degree, as done in the present work, or between in- and out-degree as shown in [19]. Furthermore, when correlations T1 and T2 were embedded in the network, single neuron deletion/stimulation of a few peculiar neurons strongly impacted the frequency of occurrence of population bursts. Most critical neurons, i.e. those capable of silencing the network when deleted or stimulated, shared common features: they constantly/reliably participated in the PB build up (i.e. they were functional hubs) and they had a quite low structural degree. These functional hubs formed a clique, where their precise ordered temporal activation was necessary for the burst generation. In the specific case here described, the clique was composed by two synaptically connected pairs, each composed of one neuron above and one below threshold. The burst could be triggered only when the first three neurons operated at precise time lags and the last neuron of the clique (which is just below threshold) was led to fire.

Each population burst caused the depletion of the synaptic resources, therefore another PB could occur only when the synaptic resources would be recovered, thus inducing an effective refractory time between two successive PBs. However, this is only a necessary, but not sufficient condition for PB triggering. The key element responsible for generating PBs was the unsupervised occurrence of a precise sequence of firing times of the two supra-threshold critical neurons, i.e. not mediated by any structural synaptic connection. On the other hand, the mode locking of the firing frequencies of these two neurons was instead responsible for *anti-resonances* associated to a drastic reduction of the PBs. For random networks, i.e. with no correlations, or embedding just one of the correlations of type T1, T2, T3 or the combination of type T1 and T3, we did not find any evidence of functional cliques and the mechanisms of network synchronization were much more robust and immune from single neuron perturbations (see Supplementary Information).

The activity of random uncorrelated networks has been previously examined in [24], in particular the authors have shown that the elimination of a pool of neurons (namely, 30 neurons, corresponding to the 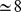
% of the excitatory population) led to the interruption of the bursting activity. The PBs were suppressed whenever the removed pool was composed by neurons with an intermediate firing rate 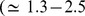 Hz). These neurons were responsible for triggering the avalanches in the network, due to their effectively strong excitatory synapses and to their proximity to the firing threshold. From these findings, it is clear that in an uncorrelated network the PBs emerge due to a cooperative effect involving a large portion of neurons. On the contrary, the introduction of correlations of type T1 and T2 induces single neuron sensitivity as discussed in this paper.

Furthermore, our results show that the integration into a clique is the key element that can enable single neurons to impact the population dynamics, without any further topological requirements for the network architecture. The functional hubs forming and operating within the clique, are actively involved in generating network synchronizations and, as a consequence, capable to impact the network dynamics when stimulated. Therefore, without necessarily being structural or effective hubs, i.e. capable to cause a direct influence on the activity of many other nodes [40], [44], they operate as *operational hubs* accordingly to the definition recently introduced in [45]. Similarly to the hub cells in the developing hippocampus whose stimulation was capable to drastically reduce the frequency of spontaneous network synchronization [5], the critical neurons presented in this paper have a very high functional connectivity and several of them are close to the firing threshold.

At variance with hippocampal hubs, critical neurons do not have a high structural degree. This is a consequence of the correlation imposed on the network where the excitability of the neurons is anti-correlated to the total structural degree of the cells. Indeed, in the correlated network studied in this work, the orchestration of the neuronal activity relies on the coordinated firing of a few critical “young” neurons (i.e. with a low structural degree) mediated by their inter-connections. However, in real biological developing networks, it is possible that a further developmental connectivity regulation is fulfilled, with the chance of finding synaptic connections in a pair of young cells much lower compared to a pair composed of a young and a mature cells. This would be also in line to the *rich gets richer* rule which can generate scale-free networks [46]. In such case, the orchestration between unconnected young neurons would require the presence of a structural connector or hub, i.e. a more developed neuron, capable to receive and promptly activate in the presence of a few synchronized inputs. Therefore, our study supports the hypothesis that, in developmentally constrained networks, PBs are triggered by a precise time activation of a few around threshold oscillators. This is indeed the case of neurons 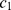 and 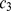, which are fundamental for the ignition of the neuronal avalanche, but they need to be activated by a precise firing sequence involving 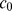 and 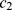. This evidence is even more striking in the example discussed in Text S3 for 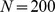 neurons, where the functional clique is composed of a small group of neurons all just below threshold, apart the leader who activates the neurons in cascade leading to the burst.

We have verified that the main ingredients required to observe strong sensitivity to single neuron stimulation and deletion are, besides the presence of the correlations of type T1 and T2, a small number of neurons supra-threshold as well as a strongly diluted network. This can find an explanation in the fact that by increasing the degree of the neurons as well as the number of neurons supra-threshold the network dynamics becomes more cooperative. Furthermore, the synaptic time scales seem not to be crucial for the emergence of single neuron sensitivity (for more details see the subsection Dependence on the Model Parameters in Methods).

Although presented within a developmental framework, our results can also have elements of interest in the wider context of brain processing. In fact, in this work we show that the introduction of an excitability gradient in the network can lead to the emergence of functional cliques capable to shape the neuronal population activity. Indeed, different brain states could dynamically modulate the level of excitability or the gain function of the neurons within a circuit (as clearly discussed in [47]) and in this way instantaneously induce the emergence of functional cliques. Furthermore, functional chains of neural activation have been reported also in the different framework of feed-forward networks [48], [49]. In this context, in Ref.[20] the authors found that structural hubs (i.e. highly connected neurons) have a peculiar role in promoting the signal transmission across sequences of non-hub sub-networks.

The previously discussed anti-resonance effect leading to the silence of the bursting activity resembles recent results reported in literature [28], where the authors have shown that abnormally synchronous neuronal populations can be desynchronized by administrating stimulations at resonant frequencies to an ensemble of spiking neurons. In that context, the desynchronization of the neuronal activity can be achieved by delivering a periodic stimulation at few sites, with a period which was an integer multiple of the fundamental period of the synchronized system. This is in striking contrast with what usually observed for a resonant forcing of a population of coupled oscillators [50], [51]. Our results, revealing population desynchronization associated to anti-resonances at the level of single neuron frequencies, are even more intriguing. On one side these findings suggest the possibility of extremely non-invasive procedures to treat pathological neuronal synchronization, which is associated to several neurological disorders [52], [53]. On the other side they reveal the potentiality of a brain circuit able to adapt to external stimuli on the basis of unsupervised mechanisms, which can switch the network activity from coherent to incoherent.

The numerical results here presented predict a primary role for supra-threshold and near-threshold cells capable to impact network synchronizations when organized into functional clique. Probing the existence of such cliques, whose emergence could be also dynamically regulated by varying the gradient of excitability in the circuits [47], is experimentally challenging, but surely feasible in *in-vitro* biological preparations. Cultured networks allow for an easier access and probing of the circuits [36], [54], while representing a general model of unsupervised (or self-organized) spontaneous network synchronization in circuits under development, analogously to what observed in central and peripheral brain circuits [55], [56]. In these circuits high functionally connected neurons (mostly activated at the build up of bursting) and highly active (i.e. supra-threshold) neurons could be identified by using both multi-electrode recordings and/or calcium imaging [54], [57]. Furthermore, by manipulating the frequency of firing of such cells through multi-site optical or electrical stimulation [21], [36] it is possible both to disrupt the sequential activation necessary for triggering network synchronizations (as displayed in Figs. 5 and 4) and to test the anti-resonance effects, as described in Fig. 8 and Table 1.

In this work, we considered a deterministic model of short term synaptic depression based on a trial-averaged representation. In recent papers, the stochastic processes involved in vesicle release and synaptic recovery time have been also taken into account to model short-term synaptic depression [58]-[60]. In particular, in Ref. [59] the authors compare deterministic and stochastic model for short-term plasticity. They found that for supra-threshold neurons the two setups give essentially the same behavior, while for sub-threshold neurons, whose spiking activity is fluctuation driven, the results of the deterministic and stochastic models essentially coincide for low frequencies (up to 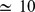 Hz). In our study the functional hubs are found to be or supra-threshold or to have a relatively low firing rate (see bottom panel in Fig. 1 C). Therefore we expect that the implementation of stochastic short-term plasticity would not affect qualitatively our main findings, but further investigations are required to fully clarify this issue.

## Methods

To study the response of bursting neural networks to single neuron stimulation and removal, we employed the Tsodyks-Uziel-Markram (TUM) model [24]. Despite being sufficiently simple to allow for extensive numerical simulations and theoretical analysis, this model has been fruitfully utilized in neuroscience to interpret several phenomena [25], [26], [32]. We have considered such a model, restricted to excitatory synapses, to somehow mimic the dynamics of developing brain circuitries, which is characterized by coherent bursting activities, such as *giant depolarizing potentials* [5], [29]. These coherent oscillations emerge, instead of abnormal synchronization, despite the fact that the GABA transmitter has essentially an excitatory effect on immature neurons [61]. The model uses leaky-integrate-and-fire (LIF) neurons with excitatory synapses displaying short-term synaptic depression [24] arranged in a directed random network. It should be stressed that we do not consider a network under topological development, which is typically characterized by a dynamical evolution (addition/deletion) of the links among the neurons.

### The model

In this paper we consider a network of 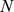 excitatory LIF neurons, interacting via synaptic currents regulated by short-term-plasticity according to the model introduced in [24]. The time evolution of the membrane potential 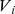 of each neuron reads as
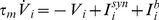
(1)where 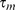 is the membrane time constant, 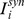 is the synaptic current received by neuron 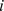 from all its presynaptic inputs and 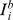 represents its level of intrinsic excitability. The membrane input resistance is incorporated into the currents, which therefore are measured in voltage units (mV).

Whenever the membrane potential 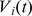 reaches the threshold value 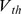, it is reset to 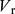, and a spike is sent towards the postsynaptic neurons. For the sake of simplicity the spike is assumed to be a 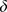
–like function of time. Accordingly, the spike-train 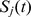 produced by neuron 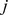, is defined as,
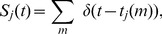
(2)where 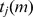 represent the 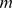-th spike time emission of neuron 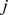. The transmission of the spike train 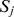 to the efferent neurons is mediated by the synaptic evolution. In particular, by following [62] the state of the synaptic connection between the 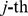 presynaptic neuron and the 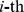 postsynaptic neuron is described by three adimensional variables, 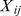, 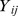, and 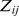, which represent the fractions of synaptic transmitters in the recovered, active, and inactive state, respectively and which are linked by the constraint 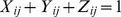. The evolution equations for these variables read as
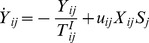
(3)

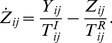
(4)


Only the active transmitters react to the incoming spikes 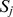: the adimensional parameters 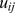 tune their effectiveness. Moreover, 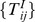 represent the characteristic decay times of the postsynaptic current, while 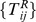 are the recovery times from synaptic depression.

Finally, the synaptic current is expressed as the sum of all the active transmitters (post-synaptic currents) delivered to neuron 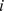


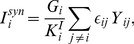
(5)where 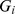 is the coupling strength, while 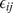 is the connectivity matrix whose entries are set equal to 1 (0) if the presynaptic neuron 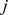 is connected to (disconnected from) the postsynaptic neuron 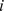. At variance with [24], we assume that the coupling strengths are the same for all the synapses afferent to a certain neuron 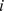. We have verified that this simplification does not alter the main dynamical features of the TUM model under control conditions.

In this paper we study the case of excitatory coupling between neurons, i.e. 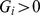. Moreover, we consider a *diluted* network made of 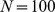 neurons where the 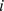-th neuron has 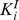 (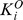) afferent (efferent) synaptic connections distributed as in a directed Erdös-Rényi graph with average in-degree 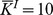, as a matter of fact also the average out-degree was 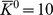. The sum appearing in (5) is normalized by the input degree 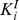 to ensure homeostatic synaptic inputs [39], [63].

The propensity of neuron 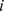 to transmit (receive) a spike can be measured in terms of the average value of the fraction of the synaptic transmitters 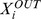 (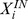) in the recovered state associated to its efferent (afferent) synapses, namely
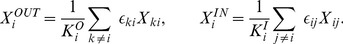
(6)


The intrinsic excitabilities of the single neurons 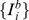 are randomly chosen from a flat distribution of width 0.45 mV centered around the value 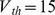 mV, with the constraint that 10% of neurons are above threshold. This requirement was needed to obtain bursting behavior in the network. This choice ensures under control conditions that the distribution of the single neuron firing rates is in the range 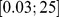 Hz.

For the other parameters, we use the following set of values: 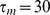 ms, 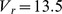 mV, 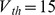 mV. The synaptic parameters 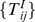, 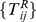, 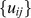 and 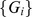 are Gaussian distributed with averages 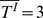 ms, 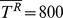 ms, 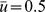 and 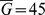 mV, respectively, and with standard deviation equal to the half of the average. These parameter values are analogous to the ones employed in [24] and have a phenomenological origin.

### Correlations

Furthermore, we have considered networks where correlations of type T1, T2 or T3 are embedded. Correlation T1 is obtained by generating randomly two pools of 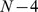 input and output degrees from an Erdös-Rényi distribution with average degree equal to 10. The degrees are ordered within each pool and then assigned to 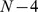 neurons in order to obtain a positive correlation between 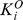 and 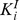. Finally, four hubs with total degree 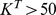 are added to this 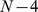 neurons. The final total degree distribution is shown in Fig. S1B.

Correlation of type T2 (T3) imposes a negative (positive) correlation between excitability 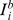 and the total degree of the single neuron 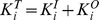. To generate this kind of correlation the intrinsic excitabilities are randomly generated, as explained above, and then assigned to the various neurons accordingly to their total connectivities 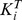, thus to ensure an inverse (direct) correlation between 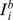 and 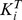. Correlations of type T2 (T3) are visualized in Fig. S1 A and Fig. S6 A.

### Numerical integration of the model

In order to have an accurate and fast integration scheme, we transformed the set of ordinary differential equations (1), (3) and (4) into an event–driven map [64] ruling the evolution of the network from a spike emission to the next one (see Text S1 for more details on the implementation of the event–driven map). It is worth to stress that the event–driven formulation is an exact rewriting of the dynamical evolution and that it does not involve any approximation.

### Population bursts

In order to identify a population burst we have binned the spiking activity of the network in time windows of 10 ms. A population burst is identified whenever the spike count involves more than 25% of the neural population. In order to study the PB build up, a higher temporal resolution was needed and the spiking activity was binned in time windows of 1 ms. The peak of the activation was used as time origin (or center of the PB) and it was characterized by more than 5% of the neurons firing within a 1 ms bin. The time window of 25 ms preceding the peak of the PB was considered as the build up period for the burst. In particular, the threshold crossing times have been defined via a simple linear interpolation based on the spike counts measured in successive time bins.

These PB definitions gave consistent results for all the studied properties of the network. The employed burst detection procedure did not depend significantly on the precise choice of the threshold, since during the inter-burst periods (lasting hundreds of milliseconds) only 10–15% of neurons were typically firing, while more than 80% of the neuronal population contributed to the bursting event (lasting 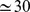 ms).

The average interburst interval for the network with (without) correlations under control conditions was 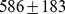 ms (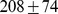 ms) for a network made of 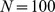 neurons, while the burst duration was 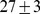 ms. Doubling the number of neurons in the correlated network did not affect particularly neither the average interburst, which became 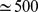 ms, nor the burst duration (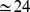 ms). For a more detailed discussion of the dynamics of this larger network see the Text S3.

### Dependence on the model parameters

In this subsection, we summarize the crucial ingredients needed to observe strong sensitivity to SNS/SND. In particular, we have checked, for different model parameters, when SNS/SND experiments were still able to noticeably modify the PB activity (i.e. more than 90% with respect to control conditions). Firstly, we considered the sparseness of the network, the reported results refer to a diluted network with a probability of 10% to have a link among two neurons. We have observed that the results of the SNS/SND experiments strongly depend on the level of dilution, however they can still be effective up to a connection probability of 50%. Another crucial aspect was the small number of neurons supra-threshold, in the studied case this number corresponded to the 10% of the neurons. By varying this percentage up to 20% we still observed that the network can be silenced by single neuron stimulation/removal. Furthermore, the dependence on the system size seemed not be crucial, since as described in Text S3 by doubling the system size a functional clique can still be identified.

We also tested the influence of the synaptic time constants on the population sensitivity to SND/SNS. As a matter of fact, the time scale ruling the depletion of the neurotransmitter 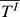 affects the duration of the PB, while the recovery time from the synaptic depression 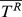 influences the intervals between consecutive PBs [24]. By varying 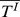 within the interval 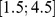 ms, while keeping fixed the ratio with 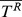, we do not observe substantial modifications on the network dynamics. Furthermore we found strong response to SNS and SND, leading to the population silence for several stimulated neurons, both for faster and slower synaptic time scale than the one actually employed in the studied example. However, it should be noticed that by increasing 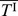 to extremely large values (namely, 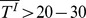 ms) this destroys the bursting behaviour in the network, which is then substituted by an asynchronous activity [43].

### Functional connectivity

In order to highlight statistically significant time-lagged activations of neurons, for every possible neuronal pair we measured the cross-correlation between their spike time series. On the basis of this cross-correlation we eventually assign a directed functional connection among the two considered neurons, similarly to what reported in [5], [54] for calcium imaging studies.

Let us explain how we proceeded in more details. For every neuron, the action potentials timestamps were first converted into a binary time series with one millisecond time resolution, where ones (zeros) marked the occurrence (absence) of the action potentials. Given the binary time series of two neurons 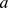 and 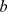, the cross correlation was then calculated as follows:
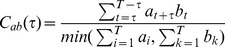
(7)where 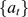,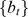 represented the considered time series and 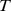 was their total duration. Whenever 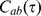 presented a maximum at some finite time value 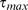 a functional connection was assigned between the two neurons: for 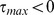 (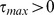) directed from 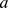 to 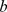 (from 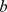 to 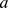). A directed functional connection cannot be defined for an uniform cross-correlation corresponding to uncorrelated neurons or for synchronous firing of the two neurons associated to a Gaussian 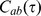 centered at zero. To exclude the possibility that the cross correlation could be described by a Gaussian with zero mean or by a uniform distribution we employed both the Student's t-test and the Kolmogorov-Smirnov test with a level of confidence of 5%. The functional out-degree 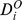 (in-degree 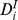) of a neuron *i* corresponded to the number of neurons which were reliably activated after (before) its firing.

#### Time series surrogates

In order to treat as an unique event multiple spike emissions occurring within a PB, different time series surrogates were defined for different kind of analysis according to the following procedures:

1. for the definition of the functional in-degree 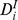 and out-degree 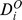, all the spiking events associated to an inter-spike interval longer than 35 ms were considered. Since we observed that this was the minimal duration of an inter-spike outside a PB and it was larger than the average duration of the PBs. This implies that for each neuron only the timestamp of the first spike within a PB was kept;
2. for the description of the PBs build up only the timestamps of the first action potential emitted within a window of 25 ms preceding the PB peak was taken into account;
3. for the analysis of the network activity during inter-burst periods, all action potentials emitted out of the PBs were considered.

### A simple model for SNS

The model here reported has been developed to reproduce the network response during the SNS experiments on 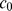 for a range of stimulation current 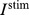, which is displayed in Fig. 3 C. To mimic this activity, we only considered the dynamics of the two neurons of the clique 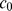 and 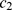 which were supra-threshold. In particular, we assumed that these two neurons fired tonically and independently as they would be two isolated LIF neurons (oscillators) [30]. Therefore, as a first step we generated two regular spike trains, one for 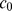 and one for 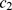 with constant inter-spike time intervals 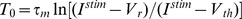 and 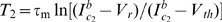, respectively. Successively, by examining the two spike trains, we assumed that a PB in the network could occur whenever the neuron 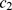 fired after 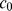 with a certain time delay 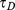. Furthermore, we also assumed that the PB emission was a probabilistic event with a finite probability 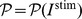. 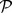 is simply given by the average efferent synaptic strength 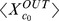 measured during the SNS experiment on 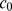 suitably rescaled in order to get probability one for 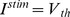. This probability has been introduced to mimic the decrease of the effective synaptic strength induced by the increasing stimulation, as shown in Fig. 6 C.

In summary, for each stimulation current 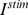 we considered the sequence of the firing times of 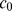 and 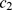 and we registered the occurrence of a PB whenever the two following conditions were both fulfilled

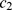
 fired after 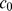 within a time window 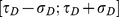, where 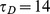 ms is the average time delay 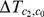 measured immediately before a population burst and 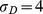 ms is its standard deviation, both measured in control conditions;
- a random number 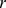 extracted by a flat random distribution with support 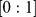 was smaller than 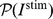.

Furthermore, each time a PB was registered, for the subsequent 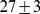 ms (corresponding to the duration of a PB under control conditions) no further PB could be counted. The results of this simple model are reported in Fig. 8 and Table 1 together with the numerical results obtained by the simulation of the network activity.

## Supporting Information

Figure S1
**Network of** 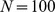 **neurons with negative correlation between** 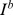 **and** 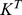 **and positive correlation between** 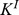 **and** 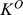 **(Setup T1 plus T2).** (A) Negative correlation between intrinsic excitability 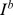 and total connectivity 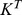. The blue dashed line indicates the threshold value 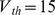 mV. (B) Positive correlation between the in-degree 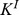 and the out-degree 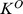. All the parameter values are defined as in Methods. The red arrows signal the neurons with the highest degrees 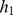, 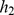, 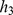. (C) Number of PBs emitted during SNS of the neurons 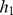, 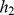, 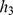 versus the stimulation current 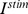. The blue vertical dashed lines mark the value of the intrinsic excitability in control condition, while the magenta horizontal solid lines indicate average number of emitted bursts under control conditions and the shaded grey area denote the amplitude of the fluctuations (measured as three standard deviations). The number of PBs are measured in all reported experiments over a time interval 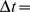 84 s.(EPS)

Figure S2
**Network of** 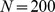 **neurons with negative correlation between** 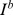 **and** 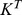 **and positive correlation between** 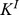 **and** 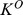 **(Setup T1 plus T2).** (A), (B) Number of PBs emitted in a time window 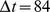 s during single neuron deletion (SND) experiments (A) and single neuron stimulation (SNS) experiments (B) with 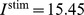 mV. The horizontal dashed lines refer to the average number of PBs emitted in a time interval 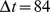 s during a control experiment when no stimulation is applied (the amplitude of the fluctuations is smaller than the symbols). Neurons are ordered accordingly to their average firing rates 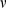 as measured during control condition (data shown in panel (C)). In the figure the green (red) circles mark the critical neurons 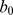, 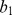, 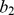, which under SND (SNS) can strongly affect the bursting activity of the network. (D) Impact on the network dynamics due to SNS of the critical neurons 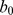, 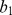, 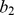 with various stimulation currents in the interval 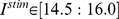 mV. The blue vertical dashed lines and the magenta horizontal solid lines mark, resp., the value of the intrinsic excitability and the bursting activity of the network during control conditions. The number of PBs are measured over a time interval 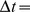 84 s. (E) Color coded rates of emission of PBs during SNS experiment performed for a range of injected DC currents 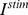 (y-axis). The PB rates during SNS are normalized to the PB rate in resting conditions. Neurons are ordered according to their functional out-degree rank (x-axis). The number of PBs are measured over a time interval 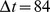 s. (F) Raster plot of the network activity: every dot denotes a firing event. The (green) dashed lines and (black) dots refer to the critical neurons. (G) Enlargement of a representative population burst: PBs are anticipated by the ordered firing sequence 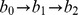. For clarity reasons, in the raster plots, at variance with all the other figures, the neuronal labels are not ordered accordingly to their firing rates.(EPS)

Figure S3
**Network of** 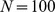 **without any correlation.** (A) Distribution of the intrinsic excitabilities 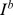 versus the corresponding total degrees 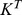. (B) Distribution of the in-degrees 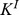 versus out-degrees 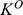 for each neuron in the network. (C) Number of population bursts, PBs, computed over the time interval 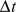  = 84 s in simulations where neurons are one by one removed by the network (SND). The horizontal dashed line refers to the average number of PBs emitted within the same time interval during the control experiment, while the shaded grey area around the line denotes the amplitude of the fluctuations (measured as three standard deviations). Here and in the following figures the data refer to 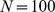 and all the parameter values are reported in Methods.(EPS)

Figure S4
**Network with positive correlation between in-degree and out-degree (Setup T1).** (A) Distribution of the intrinsic excitabilities 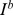 versus the corresponding total degrees 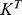. (B) Distribution of the in-degrees 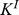 versus out-degrees 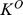 for each neuron in the network. (C) Number of population bursts, PBs, measured during SND experiments where neurons are one by one taken out from the network. (D) PBs emitted during SNS with a stimulation current 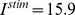 mV.(E) Single neuron frequencies 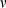 measured in a control experiment. In (C) and (D) the horizontal dashed lines and the shaded grey areas around the lines have the same meaning as in Fig. S2. All the reported data have been measured over a time window 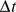  = 84 s.(EPS)

Figure S5
**Network with anticorrelation between intrinsic excitability and total degree (Setup T2).** (A) Distribution of the intrinsic excitabilities 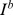 versus the corresponding total degrees 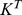. (B) Distribution of the in-degrees 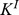 versus out-degrees 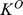 for each neuron in the network. (C) Number of population bursts, PBs, measured during SND experiments where neurons are one by one removed from the network. (D) PBs emitted during SNS with a stimulation current 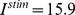 mV. (E) Single neuron frequencies 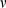 as measured during the control experiment. In (C) and (D) the horizontal dashed lines and the shaded grey areas around the lines have the same meaning as in Fig. S2. All the reported data have been measured over a time window 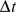  = 84 s.(EPS)

Figure S6
**Network with positive correlation between excitability and total degree (Setup T3).** (A) Distribution of the intrinsic excitabilities 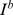 versus the corresponding total degrees 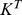. (B) Distribution of the in-degrees 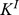 versus out-degrees 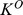 for each neuron in the network. (C) Number of population bursts, PBs, emitted during SND experiments where neurons are removed from the network one by one. (D) PBs emitted during SNS with a stimulation current 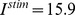 mV. (E) Single neuron frequencies 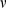 measured during a control experiment. In (C) and (D) the horizontal dashed lines and the shaded grey areas around the lines have the same meaning as in Fig. S2. All the reported data have been measured over a time window 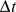  = 84 s.(EPS)

Figure S7
**Network with positive correlation between intrinsic excitability and total degree and positive correlation between in-degree and out-degree (Setup T1 plus T3).** (A) Distribution of the intrinsic excitabilities 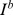 versus the corresponding total degrees 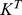. (B) Distribution of the in-degrees 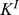 versus out-degrees 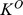 for each neuron in the network. (C) Number of population bursts, PBs, measured during SND experiments where neurons are one by one removed from the network. (D) PBs emitted during SNS with a stimulation current 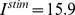 mV. (E) Single neuron frequencies 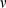 measured in control conditions. In (C) and (D) the horizontal dashed lines and the shaded grey areas around the lines have the same meaning as in Fig. S2. All the reported data have been measured over a time window 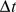  = 84 s.(EPS)

Text S1 **Event driven map.**
(PDF)

Text S2 **Dependence on different network realizations.**
(PDF)

Text S3 **Dependence on the network size.**
(PDF)

## References

[1] Wolfe J, Houweling AR, Brecht M (2010) Sparse and powerful cortical spikes. Current Opinion in Neurobiology 20: 306–312. 20400290 10.1016/j.conb.2010.03.006

[2] Brecht M, Schneider M, Sakmann B, Margrie TW (2004) Whisker movements evoked by stimulation of single pyramidal cells in rat motor cortex. Nature 427: 704–710. 14973477 10.1038/nature02266

[3] Houweling AR, Brecht M (2007) Behavioural report of single neuron stimulation in somatosensory cortex. Nature 451: 65–68. 18094684 10.1038/nature06447

[4] Cheng-yu TL, Poo Mm, Dan Y (2009) Burst spiking of a single cortical neuron modifies global brain state. Science 324: 643–646. 19407203 10.1126/science.1169957 PMC2913066

[5] Bonifazi P, Goldin M, Picardo MA, Jorquera I, Cattani A, et al (2009) Gabaergic hub neurons orchestrate synchrony in developing hippocampal networks. Science 326: 1419–1424. 19965761 10.1126/science.1175509

[6] London M, Roth A, Beeren L, Häusser M, Latham PE (2010) Sensitivity to perturbations in vivo implies high noise and suggests rate coding in cortex. Nature 466: 123–127. 20596024 10.1038/nature09086 PMC2898896

[7] Kwan AC, Dan Y (2012) Dissection of cortical microcircuits by single-neuron stimulation in vivo. Current Biology 22: 1459–1467. 22748320 10.1016/j.cub.2012.06.007 PMC3467311

[8] Barlow HB, et al (1972) Single units and sensation: a neuron doctrine for perceptual psychology. Perception 1: 371–394. 4377168 10.1068/p010371

[9] Quiroga RQ, Reddy L, Kreiman G, Koch C, Fried I (2005) Invariant visual representation by single neurons in the human brain. Nature 435: 1102–1107. 15973409 10.1038/nature03687

[10] Perrett D, Rolls ET, Caan W (1982) Visual neurones responsive to faces in the monkey temporal cortex. Experimental Brain Research 47: 329–342. 7128705 10.1007/BF00239352

[11] Beggs JM, Plenz D (2003) Neuronal avalanches in neocortical circuits. The Journal of Neuroscience 23: 11167–11177. 14657176 10.1523/JNEUROSCI.23-35-11167.2003 PMC6741045

[12] Mazzoni A, Broccard FD, Garcia-Perez E, Bonifazi P, Ruaro ME, et al (2007) On the dynamics of the spontaneous activity in neuronal networks. PloS one 2: e439. 17502919 10.1371/journal.pone.0000439 PMC1857824

[13] Petermann T, Thiagarajan TC, Lebedev MA, Nicolelis MA, Chialvo DR, et al (2009) Spontaneous cortical activity in awake monkeys composed of neuronal avalanches. Proceedings of the National Academy of Sciences 106: 15921–15926. 10.1073/pnas.0904089106 PMC2732708 19717463

[14] Hahn G, Petermann T, Havenith MN, Yu S, Singer W, et al (2010) Neuronal avalanches in spontaneous activity in vivo. Journal of Neurophysiology 104: 3312–3322. 20631221 10.1152/jn.00953.2009 PMC3007625

[15] Shriki O, Alstott J, Carver F, Holroyd T, Henson RN, et al (2013) Neuronal avalanches in the resting meg of the human brain. The Journal of Neuroscience 33: 7079–7090. 23595765 10.1523/JNEUROSCI.4286-12.2013 PMC3665287

[16] Feldt S, Bonifazi P, Cossart R (2011) Dissecting functional connectivity of neuronal microcircuits: experimental and theoretical insights. Trends in Neurosciences 34: 225–236. 21459463 10.1016/j.tins.2011.02.007

[17] Bullmore E, Sporns O (2012) The economy of brain network organization. Nature Reviews Neuroscience 13: 336–349. 22498897 10.1038/nrn3214

[18] Lee WCA, Reid RC (2011) Specificity and randomness: structure–function relationships in neural circuits. Current Opinion in Neurobiology 21: 801–807. 21855320 10.1016/j.conb.2011.07.004 PMC3223317

[19] Vasquez JC, Houweling AR, Tiesinga P (2013) Simultaneous stability and sensitivity in model cortical networks is achieved through anti-correlations between the in-and out-degree of connectivity. Frontiers in Computational Neuroscience 7: 156. 24223550 10.3389/fncom.2013.00156 PMC3819735

[20] Jahnke S, Memmesheimer RM, Timme M (2014) Hub-activated signal transmission in complex networks. Phys Rev E 89: 030701. 10.1103/PhysRevE.89.030701 24730779

[21] Wallach A, Marom S (2012) Interactions between network synchrony and the dynamics of neuronal threshold. Journal of Neurophysiology 107: 2926–2936. 22402648 10.1152/jn.00876.2011

[22] Levina A, Herrmann JM, Geisel T (2007) Dynamical synapses causing self-organized criticality in neural networks. Nature Physics 3: 857–860.

[23] Gaiteri C, Rubin JE (2011) The interaction of intrinsic dynamics and network topology in determining network burst synchrony. Frontiers in Computational Neuroscience 5: 10. 21373358 10.3389/fncom.2011.00010 PMC3044261

[24] Tsodyks M, Uziel A, Markram H (2000) Synchrony generation in recurrent networks with frequency-dependent synapses. The Journal of Neuroscience: the official journal of the Society for Neuroscience 20: 50RC+. 10.1523/JNEUROSCI.20-01-j0003.2000 PMC6774142 10627627

[25] Mongillo G, Barak O, Tsodyks M (2008) Synaptic theory of working memory. Science 319: 1543–1546. 18339943 10.1126/science.1150769

[26] Loebel A, Tsodyks M (2002) Computation by ensemble synchronization in recurrent networks with synaptic depression. Journal of Computational Neuroscience 13: 111–124. 12215725 10.1023/a:1020110223441

[27] Borkowski L (2010) Multimodal transition and stochastic antiresonance in squid giant axons. Physical Review E 82: 041909. 10.1103/PhysRevE.82.041909 21230315

[28] Lysyansky B, Popovych OV, Tass PA (2011) Desynchronizing anti-resonance effect of m: n on–off coordinated reset stimulation. Journal of Neural Engineering 8: 036019. 21555848 10.1088/1741-2560/8/3/036019

[29] Allène C, Cattani A, Ackman JB, Bonifazi P, Aniksztejn L, et al (2008) Sequential generation of two distinct synapse-driven network patterns in developing neocortex. The Journal of Neuroscience 28: 12851–12863. 19036979 10.1523/JNEUROSCI.3733-08.2008 PMC6671804

[30] Burkitt AN (2006) A review of the integrate-and-fire neuron model: I. homogeneous synaptic input. Biological Cybernetics 95: 1–19. 16622699 10.1007/s00422-006-0068-6

[31] Burkitt AN (2006) A review of the integrate-and-fire neuron model: Ii. inhomogeneous synaptic input and network properties. Biological Cybernetics 95: 97–112. 16821035 10.1007/s00422-006-0082-8

[32] Stetter O, Battaglia D, Soriano J, Geisel T (2012) Model-free reconstruction of excitatory neuronal connectivity from calcium imaging signals. PLoS Computational Biology 8: e1002653. 22927808 10.1371/journal.pcbi.1002653 PMC3426566

[33] Ben-Ari Y (2002) Excitatory actions of gaba during development: the nature of the nurture. Nature Reviews Neuroscience 3: 728–739. 12209121 10.1038/nrn920

[34] Ge S, Goh EL, Sailor KA, Kitabatake Y, Ming Gl, et al (2005) Gaba regulates synaptic integration of newly generated neurons in the adult brain. Nature 439: 589–593. 16341203 10.1038/nature04404 PMC1420640

[35] Doetsch F, Hen R (2005) Young and excitable: the function of new neurons in the adult mammalian brain. Current Opinion in Neurobiology 15: 121–128. 15721754 10.1016/j.conb.2005.01.018

[36] Marissal T, Bonifazi P, Picardo MA, Nardou R, Petit LF, et al (2012) Pioneer glutamatergic cells develop into a morpho-functionally distinct population in the juvenile ca3 hippocampus. Nature Communications 3: 1316. 10.1038/ncomms2318 PMC3535425 23271650

[37] Picardo MA, Guigue P, Bonifazi P, Batista-Brito R, Allene C, et al (2011) Pioneer gaba cells comprise a subpopulation of hub neurons in the developing hippocampus. Neuron 71: 695–709. 21867885 10.1016/j.neuron.2011.06.018 PMC3163067

[38] Karayannis T, García NVDM, Fishell GJ (2012) Functional adaptation of cortical interneurons to attenuated activity is subtype-specifc. Frontiers in Neural Circuits 6: 66. 23015781 10.3389/fncir.2012.00066 PMC3449283

[39] Turrigiano GG, Leslie KR, Desai NS, Rutherford LC, Nelson SB (1998) Activity-dependent scaling of quantal amplitude in neocortical neurons. Nature 391: 892–896. 9495341 10.1038/36103

[40] Friston KJ (1994) Functional and effective connectivity in neuroimaging: a synthesis. Human brain mapping 2: 56–78.

[41] Bullmore E, Sporns O (2009) Complex brain networks: graph theoretical analysis of structural and functional systems. Nature Reviews Neuroscience 10: 186–198. 19190637 10.1038/nrn2575

[42] Pikovsky A, Rosenblum M, Kurths J (2003) Synchronization: a universal concept in nonlinear sciences, volume 12. Cambridge University Press.

[43] di Volo M, Livi R, Luccioli S, Politi A, Torcini A (2013) Synchronous dynamics in the presence of short-term plasticity. Physical Review E 87: 032801.

[44] Sporns O (2007) Brain connectivity. Scholarpedia 2: 4695.

[45] Cossart R (2014) Operational hub cells: a morpho-physiologically diverse class of gabaergic neurons united by a common function. Current Opinion in Neurobiology 26: 51–56. 24650504 10.1016/j.conb.2013.12.002

[46] Barabási AL, Albert R (1999) Emergence of scaling in random networks. Science 286: 509–512. 10521342 10.1126/science.286.5439.509

[47] Haider B, McCormick DA (2009) Rapid neocortical dynamics: cellular and network mechanisms. Neuron 62: 171–189. 19409263 10.1016/j.neuron.2009.04.008 PMC3132648

[48] Abeles M (1991) Corticonics: Neural circuits of the cerebral cortex. Cambridge University Press.

[49] Diesmann M, Gewaltig MO, Aertsen A (1999) Stable propagation of synchronous spiking in cortical neural networks. Nature 402: 529–533. 10591212 10.1038/990101

[50] Rulkov N, Tsimring L, Larsen M, Gabbay M (2006) Synchronization and beam forming in an array of repulsively coupled oscillators. Physical Review E 74: 056205. 10.1103/PhysRevE.74.056205 17279982

[51] Kiss IZ, Zhai Y, Hudson JL (2008) Resonance clustering in globally coupled electrochemical oscillators with external forcing. Physical Review E 77: 046204. 10.1103/PhysRevE.77.046204 18517707

[52] Pare D, Curro'Dossi R, Steriade M (1990) Neuronal basis of the parkinsonian resting tremor: a hypothesis and its implications for treatment. Neuroscience 35: 217–226. 2199839 10.1016/0306-4522(90)90077-h

[53] Lenz F, Kwan H, Martin R, Tasker R, Dostrovsky J, et al (1994) Single unit analysis of the human ventral thalamic nuclear group tremor-related activity in functionally identified cells. Brain 117: 531–543. 8032863 10.1093/brain/117.3.531

[54] Bonifazi P, Difato F, Massobrio P, Breschi GL, Pasquale V, et al (2013) In vitro large-scale experimental and theoretical studies for the realization of bi-directional brain-prostheses. Frontiers in Neural Circuits 7: 40. 23503997 10.3389/fncir.2013.00040 PMC3596784

[55] Marom S, Shahaf G (2002) Development, learning and memory in large random networks of cortical neurons: lessons beyond anatomy. Quarterly Reviews of Biophysics 35: 63–87. 11997981 10.1017/s0033583501003742

[56] Blankenship AG, Feller MB (2010) Mechanisms underlying spontaneous patterned activity in developing neural circuits. Nature Reviews Neuroscience 11: 18–29. 19953103 10.1038/nrn2759 PMC2902252

[57] Eytan D, Marom S (2006) Dynamics and effective topology underlying synchronization in networks of cortical neurons. The Journal of Neuroscience 26: 8465–8476. 16914671 10.1523/JNEUROSCI.1627-06.2006 PMC6674346

[58] Goldman MS (2004) Enhancement of information transmission effciency by synaptic failures. Neural Computation 16: 1137–1162. 15130245 10.1162/089976604773717568

[59] De La Rocha J, Parga N (2005) Short-term synaptic depression causes a non-monotonic response to correlated stimuli. The Journal of Neuroscience 25: 8416–8431. 16162924 10.1523/JNEUROSCI.0631-05.2005 PMC6725676

[60] Rosenbaum R, Rubin J, Doiron B (2012) Short term synaptic depression imposes a frequency dependent filter on synaptic information transfer. PLoS Computational Biology 8: e1002557. 22737062 10.1371/journal.pcbi.1002557 PMC3380957

[61] Ben-Ari Y, Gaiarsa JL, Tyzio R, Khazipov R (2007) Gaba: a pioneer transmitter that excites immature neurons and generates primitive oscillations. Physiological Reviews 87: 1215–1284. 17928584 10.1152/physrev.00017.2006

[62] Tsodyks MV, Markram H (1997) The neural code between neocortical pyramidal neurons depends on neurotransmitter release probability. Proceedings of the National Academy of Sciences 94: 719–723. 10.1073/pnas.94.2.719 PMC19580 9012851

[63] Turrigiano GG (2008) The self-tuning neuron: synaptic scaling of excitatory synapses. Cell 135: 422–435. 18984155 10.1016/j.cell.2008.10.008 PMC2834419

[64] Zillmer R, Livi R, Politi A, Torcini A (2007) Stability of the splay state in pulse-coupled networks. Phys Rev E 76: 046102. 10.1103/PhysRevE.76.046102 17995055

