# Supplementary material for: Clique of Functional Hubs Orchestrates Population Bursts in Developmentally Regulated Neural Networks
Source: PLoS Comput Biol. 2014 Sep 25;10(9):e1003823. doi: 10.1371/journal.pcbi.1003823 (PMC4177675; doi:10.1371/journal.pcbi.1003823)
Supplement: Text S1 — Event driven map. (PDF) [file pcbi.1003823.s008.pdf]

Text S1: Event driven map

A major advantage for numerical simulations comes from the possibility of transforming the set of differential equations (1), (3) and (4) appearing in the article into an event–driven map [1]. In fact, these differential equations can be formally integrated from time $t_{\rm n}$ to time $t_{\rm n+1}$, where $t_{\rm n}$ is the instant of time immediately after the emission of the n-th spike in the network, to obtain a discrete time evolution from one spike to the successive one. The resulting map for neuron i reads

$$V_i(n+1) = V_i(n)e^{-\frac{\tau(n)}{\tau_m}} + I_i^b\left(1 - e^{-\frac{\tau(n)}{\tau_m}}\right) + G_i F_i(n) \tag{1}$$

$$Z_{ij}(n+1) = Z_{ij}(n)e^{-\frac{\tau(n)}{T^R_{\rm ij}}} + \frac{T^R_{\rm ij}}{T^R_{\rm ij} - T^I_{\rm ij}} Y_{ij}(n)\left(e^{-\frac{\tau(n)}{T^R_{\rm ij}}} - e^{-\frac{\tau(n)}{T^I_{\rm ij}}}\right) \tag{2}$$

$$Y_{ij}(n+1) = Y_{ij}(n)e^{-\frac{\tau(n)}{T^I_{\rm ij}}} + u_{ij}\left[1 - \frac{T^R_{\rm ij}}{T^R_{\rm ij} - T^I_{\rm ij}} Y_{ij}(n)\left(e^{-\frac{\tau(n)}{T^R_{\rm ij}}} - \frac{T^I_{\rm ij} e^{-\frac{\tau(n)}{T^I_{\rm ij}}}}{T^R_{\rm ij}}\right) - Z_{ij}(n)e^{-\frac{\tau(n)}{T^R_{\rm ij}}}\right]\delta_{i,s}, \tag{3}$$

where the index s refers to the neuron spiking at time t_{n+1}, $\tau(n) = t_{n+1} - t_n$ is the n-th inter–spike–interval (ISI) in the network and $F_i(n)$ has the following expression,

$$F_i(n) = \frac{1}{K_i^I}\sum_{j \neq i} \epsilon_{ij} Y_{ij}(n) \frac{T^I_{\rm ij}}{T^I_{\rm ij} - 1}\left(e^{-\frac{\tau(n)}{T^I_{\rm ij}}} - e^{-\frac{\tau(n)}{\tau_m}}\right), \tag{4}$$

with the sum running over the index j, which denotes all direct connections (afferent synapses) reaching neuron i from all the other neurons. Notice that $\tau(n)$ can be determined by computing the time

$$\tau_i(n) = \ln\left[\frac{I_i^b - V_i(n)}{I_i^b + G_i F_i(n) - 1}\right],\ i = 1, \cdots N\,; \tag{5}$$

needed to each neuron ith neuron to reach the threshold value and by selecting the shortest one, namely

$$\tau(n) = \inf_i\{\tau_i(n) | i = 1, 2, \cdots, N\}.$$

References

1. Zillmer R, Livi R, Politi A, Torcini A (2007) Stability of the splay state in pulse-coupled networks. Phys Rev E 76: 046102.
