# Supplementary material for: Clique of Functional Hubs Orchestrates Population Bursts in Developmentally Regulated Neural Networks
Source: PLoS Comput Biol. 2014 Sep 25;10(9):e1003823. doi: 10.1371/journal.pcbi.1003823 (PMC4177675; doi:10.1371/journal.pcbi.1003823)
Supplement: Text S2 — Dependence on different network realizations. (PDF) [file pcbi.1003823.s009.pdf]

Text S2: Dependence on different network realizations

We repeated the SNS/SND experiments for eight different realizations at $N = 100$ of the intrinsic excitability and of the synaptic parameters as well as of the connectivity matrix. In all the performed experiments the parameter values are taken from random distributions with the same averages and standard deviations as reported in Methods. Furthermore, the networks have been realized with the same average in-degree and the same constraints described in Methods. In five of the examined cases we found a response to SNS/SND experiments qualitatively similar to the one examined in the paper. In particular, we have identified a number of critical neurons ranging from 6 to 3 in each realization with associated intrinsic excitability in the range [14.82 : 15.30] mV, i.e. slightly below or above the firing threshold. In most of the cases the critical clique contained two neurons supra-threshold, as in the example discussed in the paper. Furthermore, in four cases the stimulation/deletion of the critical neurons lead to the total suppression of the bursting activity (within the considered time interval), while in one case we observed very strong reductions of the population bursts (up to 65% of the activity recorded in control conditions).
