# Supplementary material for: Clique of Functional Hubs Orchestrates Population Bursts in Developmentally Regulated Neural Networks
Source: PLoS Comput Biol. 2014 Sep 25;10(9):e1003823. doi: 10.1371/journal.pcbi.1003823 (PMC4177675; doi:10.1371/journal.pcbi.1003823)
Supplement: Text S3 — Dependence on the network size. (PDF) [file pcbi.1003823.s010.pdf]

Text S3: Dependence on the network size

In order to test if the size had any influence in the observed phenomena, we considered a directed random graph with $N = 200$ neurons and an average in-degree $\bar{K}^I = 10$ and with the embedded correlations of type T1 and T2. The network activity was still characterized by bursts of duration $\simeq 24$ ms and with interburst intervals $\simeq 500$ ms, thus suggesting that the doubling of the size had not altered the main dynamical features of the network.

We performed SND and SNS experiments also in the present case. SND experiments revealed that the removal of 3 critical neurons can lead to the complete silence of the network, these neurons are identified in Fig. S2 by the labels b_0, b_1 and b_2. As shown in Fig. S2 B, the SNS experiments, performed with stimulation current $I^{\rm stim} = 15.45$ mV, confirmed the capability of neurons b_1 and b_2 to silence the network, while SNS on neuron b_0 reduced the PBs of $\simeq 27\%$. The most critical neurons were all unable to fire if isolated from the network, i.e. they were all below threshold, apart neuron b_0, which had $I^b_{b_0} = 15.19$ mV.

An extensive investigation of the critical neurons subjected to stimulations with currents in the range $I^{\rm stim} \in [14.5 : 16.0]$ mV revealed that PBs can be observed only if the neurons b_1 and b_2 had excitabilities within a narrow range (of amplitude $\simeq 0.2$ mV) centered around the threshold value. While, the stimulation of neuron b_0 revealed an absolute minimum in the PB activity (an *anti-resonance*) at $I^{\rm stim} = 15.32$ mV and a relative minimum at $I^{\rm stim} = 15.45$ mV (see Fig. S2 D). We performed also an extensive analysis of the response under SNS experiments for all the neurons of the network. As it is summarized in Fig. S2 E, we found only sporadic significant reduction in the number of PBs occurring in extremely narrow current intervals.

Therefore also for $N = 200$ we observed that SND or SNS were capable to silence the network and that this occurred only for a quite limited number of neurons, which had low K^T and reasonably high I^b.

The detailed investigation of the burst events revealed that each PB was always preceeded by the firing of the 3 critical neurons in the following order: $b_0 \rightarrow b_1 \rightarrow b_2 \rightarrow$ PB, as it is shown in Fig. S2 FG. The neuron b_0, which was the only one supra-threshold, fired first followed by the others (sub-threshold) and this triggered the onset of the PB. As shown in Fig. S2 D, whenever b_1 and b_0 were stimulated with currents $I^{stim} > I^b_{b_0} = 15.19$ mV the bursting activity stopped. This behaviour is analogous to what reported for the smaller network, indicating that neuron b_0 is the leader and the other ones are simply followers in the construction of the PB, they cannot fire more rapidly then neuron b_0 or the PBs ceased. An analysis of the structural connectivity revealed that neuron b_0 projected an efferent synapse on b_1, which projected on b_2. We can safely affirm, also in this case, that neurons b_0, b_1 and b_2 form a functional clique, whose sequential activation is essential for the population burst onset.
